# Economic evaluation of novel *Mycobacterium tuberculosis* specific antigen-based skin tests for detection of TB infection: A modelling study

**DOI:** 10.1371/journal.pgph.0002573

**Published:** 2023-12-20

**Authors:** Lara Goscé, Kasim Allel, Yohhei Hamada, Alexei Korobitsyn, Nazir Ismail, Saima Bashir, Claudia M. Denkinger, Ibrahim Abubakar, Peter J. White, Molebogeng X. Rangaka

**Affiliations:** 1 Institute for Global Health, University College London, London, United Kingdom; 2 Department of Infectious Disease Epidemiology, London School of Hygiene and Tropical Medicine, London, United Kingdom; 3 Department of Disease Control, London School of Hygiene and Tropical Medicine, London, United Kingdom; 4 Unit for Prevention, Diagnosis, Treatment, Care and Innovation, Global Tuberculosis Programme, World Health Organization, Genève, Switzerland; 5 Division of Infectious Diseases and Tropical Medicine at University Hospital Heidelberg, Heidelberg, Germany; 6 German Center for Infection Research, Heidelberg University Hospital, Heidelberg, Germany; 7 MRC Centre for Global Infectious Disease Analysis and NIHR Health Protection Research Unit in Modelling and Health Economics, School of Public Health, Faculty of Medicine, Imperial College, London, United Kingdom; 8 Modelling and Economics Unit, UK Health Security Agency, London, United Kingdom; University of Ottawa, CANADA

## Abstract

Evidence on the economic impact of novel skin tests for tuberculosis infection (TBST) is scarce and limited by study quality. We used estimates on the cost-effectiveness of the use of TBST compared to current tuberculosis infection (TBI) tests to assess whether TBST are affordable and feasible to implement under different country contexts. A Markov model parametrised to Brazil, South Africa and the UK was developed to compare the cost-effectiveness of three TBI testing strategies: (1) Diaskintest (DST), (2) TST test, and (3) IGRA QFT test. Univariate and probabilistic sensitivity analyses over unit costs and main parameters were performed. Our modelling results show that Diaskintest saves $5.60 and gains 0.024 QALYs per patient and $8.40, and 0.01 QALYs per patient in Brazil, compared to TST and IGRA respectively. In South Africa, Diaskintest is also cost-saving at $4.39, with 0.015 QALYs per patient gained, compared to TST, and $64.41, and 0.007 QALYs per patient, compared to IGRA. In the UK, Diaskintest saves $73.33, and gaines 0.0351 QALYs per patient, compared to TST. However, Diaskintest, compared to IGRA, showed an incremental cost of $521.45 (95% CI (500.94–545.07)) per QALY, below the willingness-to-pay threshold of $20.223 per QALY. Diaskintest potentially saves costs and results in greater health gains than the TST and IGRA tests in Brazil and South Africa. In the UK Diaskintest would gain health but also be more costly. Our results have potential external validity because TBST remained cost-effective despite extensive sensitivity analyses.

## Introduction

Tuberculosis (TB) continues to threaten population health causing a substantial burden of disease [1]. However, a reduced access to TB diagnosis and treatment has resulted in an increase in TB deaths, specifically after the disruption on TB services triggered by the COVID-19 pandemic [2]. The World Health Organisation (WHO) TB 2022 report estimated 1.6 million TB deaths occurred in 2021 including a large reduction on the number of people receiving preventive treatment and being diagnosed, increase of 23% with respect to 2020 [3]. Mitigating and reversing these impacts are required. Provision of new TB services, including early testing for detection of TB, are essential to lower the current TB burden, especially in most affected countries and populations. Current standards for TB infection (TBI) diagnosis recommend TB skin test (TST) using purified protein derivative (PPD) and interferon gamma release assay (IGRA), such as QuantiFERON-TB test (QFT) or T-SPOT.TB, as tests to identify TB infection, preventing further TB-related burden [4]. However, which is preferable remains unclear and is highly dependent on countries resources and population characteristics. The advantages of IGRA includes higher specificity and sensitivity and the need for only one contact with a healthcare professional [5]. However, IGRA implementation and scale up can be expensive in resource-constrained settings. The TST, on the other hand, may offer an affordable alternative to IGRA, but it requires two clinical visits within 2–3 days of testing which might be expensive and unfeasible for those individuals having limited access to healthcare.

Intermittent shortage of these tests, low specificity in specific populations [6], and the vast staff training needed to employ these tests, has highlighted the necessity for exploring the adoption of newer tests [7]. However, economic assessments of the feasibility of a newer test recommended by the WHO (i.e. TSBT), compared to the most clinically accepted tests, has not yet been evaluated.

**Fig 1:**
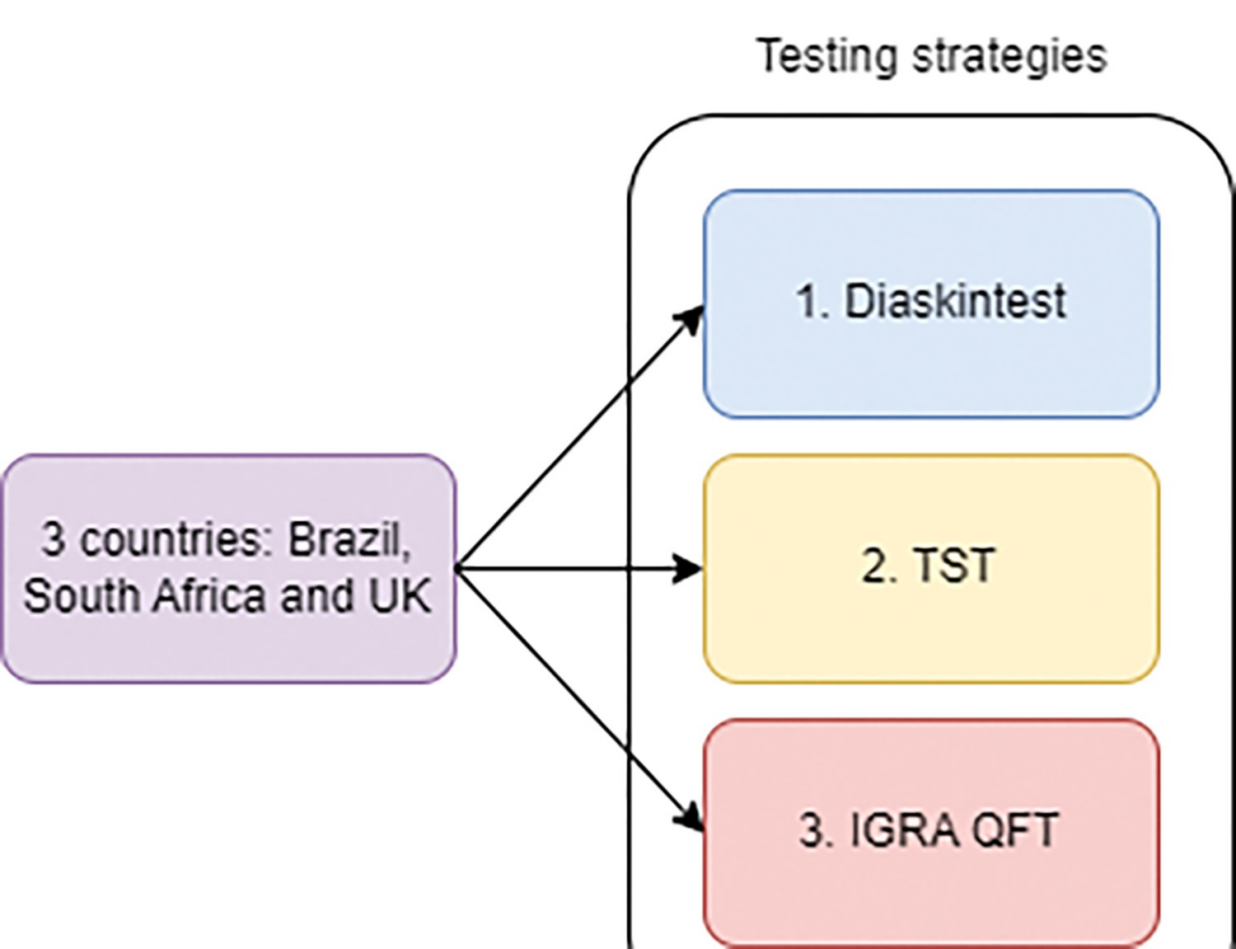
Flow diagram showing the 3 different screening strategies in the 3 countries.

The present study aims to model the cost-effectiveness of TBST (strategy 1), compared to TST test (strategy 2) and current IGRA (strategy 3) (Fig 1), by simulating a cohort of individuals being offered TBI testing. We analyse whether the test uptake has an effect in reducing TB in three different country settings (low-, middle- and high-income countries) to account for different population characteristics and costing schemes: South Africa, Brazil and the UK.

## Material and methods

### Model scheme and characteristics

We developed a Markov model simulating a cohort of individuals transitioning among different states for individuals without active TB at first stage. Following the cascade of care, these individuals are tested for TB infection (TBI), if positive, they may initiate and eventually complete or interrupt treatment. Fig A in S1 Text document depicts the full model scheme and its states: (1) no tuberculosis infection, tested; (2) tuberculosis infection, tested; (3) no tuberculosis infection, treated; (4) no tuberculosis infection, untreated; (5) no tuberculosis infection, treatment started but interrupted; (6) tuberculosis infection, treated; (7) tuberculosis infection, untreated; (8) tuberculosis infection, treatment started but interrupted; (9) active TB; (10) no TB; (11) death. The model is parametrised to three settings reflecting different TB burden and country income: United Kingdom, Brazil and South Africa.

### Model parameters

The probabilities of moving between states are presented in Table 1. We initialise the model by assuming 100,000 people without active TB (of all ages) get tested for TB infection. All parameters are sourced from the literature including tests’ specificity and sensitivity. TBST specificity is currently assumed to be equal to that of the IGRA (QuantiFERON-TB or T-SPOT.TB [8]). We used a population-based approach integrating all individuals having a recent TBI, including TB case contacts, immunocompromised populations, among other vulnerable groups.

**Table 1:** Model parameters.

Parameter	Value	Source
Prevalence of TB infection in TB-negative individuals, percentage	Brazil = 13.27% [95%CI = 9.4–18.1] UK = 1.76% [95%CI = 1.3–2.7] South Africa = 31.53% [95%CI = 28.8–36.1]	Literature [9] (see Table A in S1 Text)
People completing treatment after initiation following a positive TBI result, percentage	Brazil = 33.96% UK = 44.2% South Africa = 12.06%	Brazil [10] UK, NHS [11] South Africa [12]
People not initiating treatment after testing positive for TBI, percentage	Brazil = 39.62% UK = 36% South Africa = 27.8%	Brazil [10] UK, NHS [11] South Africa [12]
People whose treatment was interrupted after initiation following a positive TBI test result, percentage	Brazil = 26.42% UK = 19.8% South Africa = 60.1%	Brazil [10] UK, NHS [11] South Africa [12]
Progression (evolution) from TBI to Active TB, probability	0.08 [95%CI = 0.05–0.10]	[13–15]
Efficacy of TBI treatment	90% (63–93%)	[16]
Active TB treatment coverage	Brazil = 78% [95%CI = 67–91] UK = 89% [95%CI = 81–98] South Africa = 58% [95%CI = 43–83]	[17]
Recovery from Active TB (treated+untreated)	Brazil = 59.1% UK = 71.7% South Africa = 54.6%	[17, 18]
Death from Active TB (treated+untreated)	Brazil = 11.1% [95%CI = 9.1–12.7] UK = 14.1% [95%CI = 10.3–16.4] South Africa = 9.4% [95%CI = 8.0–10.6]	[17, 18]
Probability of a true positive test result if the patient has TB infection (sensitivity)	**TST:** 88.24 (78.20–94.01) **Diaskintest:** 91·18 (81.72–95.98) **IGRA test (QFT):** 89.66 (78.83–95.28) **IGRA test (T-SPOT):** 90.91 (79.95–96.16) **Cy-TB test (skin):** 86·06 (82.39–89.07)	TBST systematic review [5]
Probability of a true negative test result if the patient does not have TB infection (specificity)	**TST**: 93.31 (90.22–95.48) **Diaskintest:** 99.15 (79.66–99.97) **IGRA test (QFT):** 99.15 (79.66–99.97) **Cy-TB test (skin):** 97.85 (93.96–99.25)	TBST systematic review [5]. Diaskintest’s specificity is assumed to be equal to IGRA (QFT) specificity

*Notes*: IGRA = interferon-γ release assays. QFT = QuantiFERON-TB Gold. TST = tuberculin skin test. Differentiation between HIV status or time since infection are not included in the model but Table B in S1 Text shows how the parametrisation of the model could be modified to account for different population progression rates and infection duration.

### Costs per test unit and utility scores

#### Test costs and screening strategies schemes

Table 2 displays unit costs and utility scores, having costs expressed in 2021 United States Dollars (USDs). IGRA and TST unit costs were sourced from a recent systematic literature review [19]. These costs included test kit, staff time, and disposable and laboratory costs. Market value of Diaskintest costs were provided by the manufacturer and reported in Tables D, E in S1 Text. To calculate the unit cost for Diaskintest, the same extra costs resulting from the TST systematic review (such as staff time, disposable, and laboratory costs) were added to the test cost provided by the manufacturer. We incorporated the most conservative ‘largest’ possible test costs and according to each country’s prescribed screening strategy. Three testing strategies were identified. First, the new TBST strategy using Diaskintest. Second, the TST skin test strategy using PPD. Third, the QFT-plus strategy which uses one millimetre of aliquots of whole blood and it is incubated with antigens overnight, according to the manufacturer guidelines [20]. Specifically for the UK’s TBI testing, we extracted full test costs (including follow-up visits) according to the National Health System (NHS) tariffs and in line with NICE guidelines [21, 22]. TST is required to have two clinic visits while it is one for IGRA (T-SPOT.TB or QFT-GIT). For South Africa, we followed a full screening strategy that comprised test costs (disposables, administration, reading, laboratory technicians), two clinic visits, and one chest radiograph plus one outpatient laboratory visit for IGRA tests [23]. For Brazil, we sourced costs from Steffen *et al*. (2020) [16] which however only included the cost of the test, staff time, and consumables.

**Table 2:** Unit costs and utility parameters.

Test unit costs or utility value (unit costs/utilities)	Value	Source
Diaskintest cost		
Brazil	$ 5.33^a^	(see Tables D, E in S1 Text)
South Africa	$ 90.60^a^^,^^b^	
United Kingdom	$ 181.43^a^^,^^c^	
TST test cost		
Brazil	$ 7.66	[24]
South Africa	$ 99.13^b^	[23]
United Kingdom	$ 181.63^c^	[21]
IGRA test (QFT) cost		
Brazil	$22.17	[24]
South Africa	$220.02^b^	[23]
United Kingdom	$149.40^c^	[21]
Utility scores		
Utility without TB (normal health)	0.88	
Utility loss due to untreated active TB	0.19	
Utility loss associated with inpatient treatment	0.210	[18]
Utility loss associated with outpatient treatment	0.067	
Utility loss due to active TB treatment adverse effects	0.17	
Utility loss due to TBI treatment	0.2	

*Notes*: Costs provided in 2021 USDs.

^a^Unit cost calculated by summing Diaskintest costs as provided by the manufacturer (Tables D, E in S1 Text) and TST associated costs (excluding test cost) from literature review [21, 23, 24].

^b^These costs represent the whole screening strategy including the costs of the tests (disposables, administration, reading, laboratory technicians), two clinic visits and one chest radiograph. Screening strategies that include an IGRA also include the cost of one outpatient laboratory visit. The unit cost for TST (including only disposables, administration, reading, laboratory technicians) is $21.92 without the two clinic visits and chest radiograph. Cost of TST test only is sourced from Laskin et al. (2013) [40] as $8.53.

^c^Abubakar et al. assumes that test strategies involving TST would require two clinic visits, whereas those involving only IGRAs (T-SPOT.TB or QFT-GIT or both) would only require one clinic visit.

#### TBI treatment costs

Unit costs per TBI treatment are provided in Table 3. Costs were calculated by summing the cost of initial single medical consultation with monthly follow-ups. The number of follow-up consultations depends upon the regimen (i.e. three months of treatment with isoniazid (300 mg/day) (3H):2; 6H: 5; 9H: 8; 12H: 11). UK costs are calculated using NHS tariffs. Costs for TB disease treatment for a directly observed therapy (DOT) approach (active TB) can also be found in Table 3, by regime and country.

**Table 3:** Costs per treatment, by country (inflated, expressed in 2021 USDs).

**Cost per TBI treatment regime, by country**		**Drug costs**		**Staff costs (medical and nurse follow-up consultations)**		**Total costs**	
Brazil [24]							
3H		45.8		16.4		62.2	
6H		91.7		32.8		124.4	
9H		137.5		49.1		186.7	
12H		183.3		65.5		248.9	
South Africa [25]							
3H		3.1		2.9		6.0	
6H		6.2		5.6		11.8	
9H		9.3		8.2		17.5	
12H		12.4		10.9		23.3	
United Kingdom [26]							
3H		200.7		294.6		684.7	
6H		395.6		517.4		1,261.9	
9H		790.0		740.1		2,115.0	
12H		803.9		962.8		2,442.1	
**Costs per active TB treatment (6 months treatment)**	**Drug costs**		**Staff costs (medical and nurse follow-up consultations)** ^**a**^		**Chest X-ray, culture tests, and liver function tests costs** ^**h**^		**Total cost per case**
Brazil							
2 months RHZE	$16.7 [27]						
4 months RH	$19.09 [27]		$332.3 ^b^		$41.93 ^e^		$410.52
6 months treatment	$35.79						
South Africa							
2 months RHZE	$20.72 [28]						
4 months RH	$25.90 [28]		$64.68 ^c^		$221.53 ^f^		$332.83
6 months treatment	$46.62						
United Kingdom							
2 months RHZE	$195.09 [29]						
4 months RH	$206.91 [29]		$7,653.9 ^d^		$180.67 ^g^		$8,236
6 months treatment	$402						

*Notes*: Values were inflated in their respective currencies and then exchanged into 2021 USD, if corresponded.

Average exchange rate in 2021 between £ and $ was used (£ = 1.3823 USD$). More details provided in Table C in S1 Text. 3H = three months of treatment with isoniazid (300 mg/day), 6H = 6 months treatment with isoniazid, 9H = = 9 months treatment with isoniazid, 12 = 12 months treatment with isoniazid. R—Rifampicin, H—Isoniazid, Z—Pyrazinamide, E–Ethambutol

^a^ Calculated considering 5 weekly visits during the intensive phase (first 2 months) and twice weekly during continuation phase (last 4 months) administered by nurses for a six-month treatment, plus two medical consultations based on the WHO [30]

^b^ Single cost per medical consultation = 2020 $4.3 and single cost Follow-up check = 2020 $4.3 [24].

^C^ Single cost per medical consultation = 2016 $0.98, Single cost Follow-up check = 2016 $0.77 [25]

^d^ 2015, Single cost per medical consultation = £126, Single cost Follow-up check = £64 [26].

^e^ Brazil [13]: Culture test = $2.39, Chest X-ray = $4.04, Liver function test = $3.5.

^f^ South Africa [28]: Culture test = $14.02, Chest X-ray = $31.91, Liver function test = $15.05.

^g^ UK[29]: Culture test = $10, Chest X-ray = $28, Liver function test = £1

^h^ We considered 4 cultures, 4 liver function tests, and 2 chest-X rays per case treated as per UK guidelines [29]. DOT: Directly observed therapy.

#### Utility scores

Cost per QALY is calculated based on utility scores associated to TBI treatment (Table 2). We assume that TB infection causes no utility loss, however treating TBI averts active TB, which averts mortality due to active TB, morbidity due to active TB, and side effects of active TB treatment. We also considered QALY losses due to the side effects of treating TBI.

### Statistical analysis

We perform an economic evaluation by contrasting and comparing three testing alternatives: (i) Diaskintest; (ii) TST test; and (iii) IGRA, QuantiFERON-TB test (either Gold In-Tube or Gold-Plus). Our model was parametrised for each of the three selected countries and testing strategies. Results from the Diaskintest strategy (strategy 1) were compared against TST (strategy 2), and IGRA (strategy 3). Our results were estimated over a time horizon of 20 years, we used a discount rate of 3.5% in the UK, 5% in Brazil and 3% in South Africa as per standard practice [16, 21, 23]. Results are presented as incremental cost-effectiveness ratios (ICERs) or incremental net benefit (INB) where appropriate. We compared the ICERs calculated with a country-specific willingness to pay threshold value per QALY that was sourced from the literature [31]. If the intervention was cost saving, we calculated the INB. A QALY is valued at $2784-$8755 in Brazil, $1367-$5783 in South Africa, and $23535 in the UK [31, 32].

#### Sensitivity analyses

To account for parameter uncertainty, we performed a probabilistic sensitivity analysis employing a Monte Carlo simulation with 1,000 iterations. The uncertainty in clinical probabilities, accuracies and utilities were assumed to have a beta distribution, while a gamma distribution was assumed for costs, with a 20% uncertainty boundary if not otherwise stated. Moreover, univariate sensitivity analysis was also performed on TBST unit costs to identify a possible maximum value for TBST to remain cost saving or cost-effective where appropriate.

All statistical analyses were done in **R** software, version 16, 2022.

## Results

### Brazil

**Fig 2:**
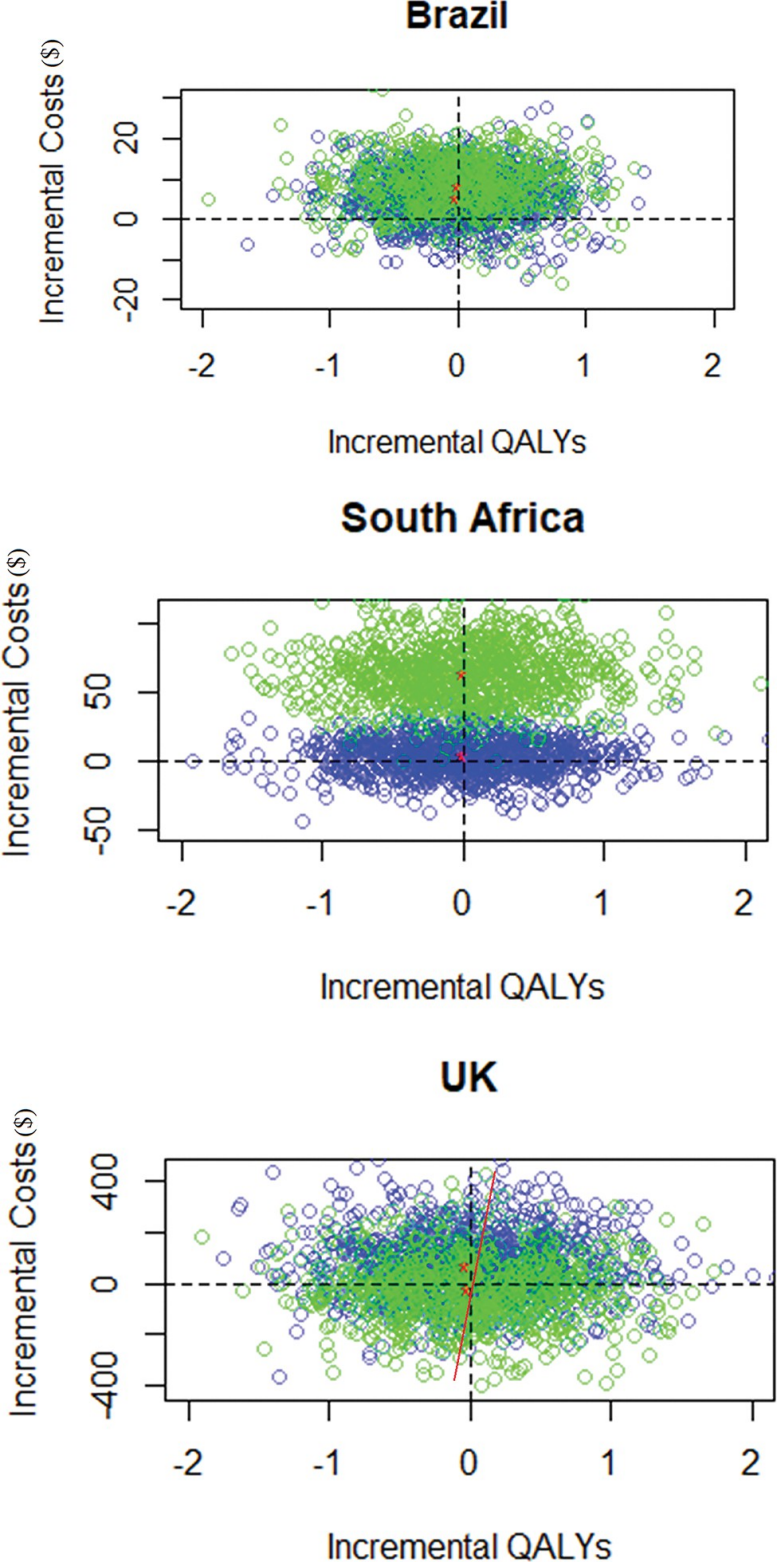
Incremental cost-effectiveness planes of Diaskintest versus TST (blue), and Diaskintest versus IGRA (green), in Brazil, South Africa and UK.

Strategy 1 was cost saving compared to both strategy 2 and 3 (Fig 2). Compared to TST, Diaskintest was cost-saving at $5.60 with an incremental gain of 0.024 QALYs per patient. Compared to IGRA, Diaskintest was cost saving at $8.40 with an incremental gain of 0.01 QALYs per patient. As the intervention was cost saving, we calculated INB rather than ICER (Table 4). Sensitivity analysis on cost of the test (Table F in S1 Text) shows that, assuming same sensitivity and specificity values as Diaskintest, a testing strategy using any TBST would remain cost saving compared to TST for an increase in test’s unit cost of up to approximatively two times the unit cost of Diaskintest. Meaning, a test unit cost of $16.06 would lead to an average expected cost per patient equal to TST’s ($35.45).

**Table 4:** Modelled costs and effects of the three strategies in Brazil, South Africa and UK. Base case (mean of 1,000 PSA iterations).

Strategy	Cost ^a^	QALY ^a^	Incremental Cost (vs Diaskintest)	Incremental QALY (vs Diaskintest)	INB
*Brazil*					
(i) Diaskintest	29.84	11.12065			
(ii) TST	35.45	11.09708	5.61	− 0.02357	− 183.42
(iii) IGRA	38.26	11.11064	8.43	− 0.01001	− 83.95
*South Africa*					
(i) Diaskintest	60.50	13.1155			
(ii) TST	64.89	13.10031	4.39	− 0.0152	− 35.03
(iii) IGRA	124.91	13.10905	64.41	− 0.0065	− 95.05
*United Kingdom*					
(i) Diaskintest	640.71	12.83467			
(ii) TST	714.04	12.79959	73.33	− 0.0351	− 783.16
(iii) IGRA	624.91	12.8044	− 15.80	− 0.0303	− 628.56

*Notes*: Positive incremental costs indicate the amount of money saved on average, when using Diaskintest instead of TST or IGRA. Negative incremental costs indicate the amount of money lost when using Diaskintest. Similarly, negative incremental QALYs indicate that strategies using TST or IGRA comport a loss in QALYs compared to Diaskintest strategy.

^a^ Mean per patient (discounted).

QALY: quality-adjusted life years, PSA: Probability sensitivity analysis, INB: Incremental net benefit.

### South Africa

Strategy 1 was cost saving compared to both strategy 2 and 3 (Fig 2). Compared to TST, Diaskintest was cost-saving at $4.39 with an incremental gain of 0.0152 QALYs per patient. Compared to IGRA, Diaskintest was cost saving at $64.41 with an incremental gain of 0.0065 QALYs per patient. As the intervention was cost saving, we calculated INB rather than ICER (Table 4). Sensitivity analysis on cost of the test (Table F in S1 Text) shows that, with same sensitivity and specificity values as Diaskintest, the Diaskintest would remain cost saving compared to TST for an increase in test’s unit cost of up to $9 compared to the unit cost of Diaskintest. Meaning, a test unit cost of $99.93 would lead to an average expected cost per patient equal to TST’s ($64.89).

### United Kingdom

Strategy 1 was cost saving against strategy 2 but not compared with strategy 3 (Fig 2). Compared to TST, Diaskintest was cost saving at $73.33 with an incremental gain of 0.0351 QALYs per patient. Compared to IGRA, Diaskintest showed an incremental cost of $15.80 and an incremental gain of 0.0266 QALYs per patient, or $521.45 (95% CI (500.94 – 545.07)) per QALY, below the willingness-to-pay threshold of $21,453 per QALY [31]. Sensitivity analyses on the cost of the test (Table F in S1 Text) show that, with same sensitivity and specificity values as Diaskintest, a TBST strategy would become cost saving compared to IGRA for a decrease in the unit cost of the test to approximatively the same unit cost as IGRA. Meaning, a test unit cost of $149 would lead to an average expected cost per patient equal to TST’s ($624.91). Moreover, a strategy with a test unit cost up to $426 (i.e. 2.35 times Diaskintest’s unit cost) would still be considered cost-effective when compared to the willingness-to-pay threshold of $21,453 per QALY [31].

## Discussion

Our modelling results show that Diaskintest potentially dominates both TST and IGRA in Brazil and South Africa, as it saves costs and gains health. In the UK Diaskintest gains more health than TST and IGRA, and is cheaper than TST, but it is more expensive than IGRA–although the health gain means that the ICER would be considered cost-effective. Moreover, IGRA is more costly than TST but gains more health in Brazil and South Africa, while IGRA is cheaper and more effective than TST in the UK. We also performed a univariate sensitivity analysis on TBST unit costs and compared the results of the three strategies, identifying possible maximum unit costs of new TBST for the strategy to remain cost-saving or cost-effective even if costs are doubled in Brazil and South Africa; countries among the top 30 TB (and HIV-TB) burden worldwide [33].

Considering currently available tests, cheaper and safer tests and treatments are necessary to account for a broader population, especially in highly endemic TB populations, to enhance deployment and acceptance of tests while avoiding treatment dropout. TBST has similar accuracy to IGRA’s [5], and cheaper costs compared with TST. Diaskintest was incrementally cost-saving between $4.4 and $61.4 in Brazil and South Africa, compared with either TST or IGRA tests. The IGRA test was more expensive but similarly effective. In contrast, Diaskintest and IGRA subjugated TST due to its reduced accuracy when identifying potential subjects for TBI treatment (false-positive screen results because of low test specificity) [34]. In the UK, Diaskintest is cost-saving at $73.3 compared with TST, but it is more costly when contrasted with IGRA, according to our study results. IGRA tests are the most cost-effective strategy within the UK, and National guidance recommends the usage of IGRA or a dual strategy TST-IGRA combined [35, 36]. This might be primarily attributed to its decreased direct costs calculated and the high effectiveness in diagnosing TBI (averting TB cases). However, the TBST test is more expensive in the UK due to non-existing test providers locally and the high transportation costs estimated by the manufacturer supplier. Furthermore, we exhibited modest but positive incremental health gains for Diaskintests in every country (ranging from 0.01 to 0.04 QALYs), which comprises a consistent measure in economic evaluations for TBI diagnosis [19, 34, 37, 38]. The small resulting values exhibit the significant number of individuals to treat to avert one case of TBI augmented by the negligible impact on QALYs due to existing health risks and treatment. A parallel study displayed incremental health gains of 0.001 and 0.03 QALYs for Diaskintest, compared to IGRA (QFT) and TST, respectively [16]. Consequently, our estimates are aligned with previous findings.

Multiple factors affecting the analysis need to be carefully considered when interpreting results. First, the costs of Diaskintest were provided by the manufacturer, accounting for delivery volume and including delivery costs for each setting. We assumed the same volume of the test for the three countries in our study to account for 100,000 individuals tested, however, this figure will most likely vary according to the country’s needs. Reported data indicate that larger orders come with smaller delivery fees. Moreover, the manufacturer reported that one vial (which serves 15 patients) could be used for up to two hours, leading to wasted doses and, consequently, increased costs per patient. However sensitivity analysis on number of tests showed that in the worst case scenario of only one test performed with one vial, Diaskintest was still cost-effective (but no longer cost saving in Brazil and South Africa, see Table G in S1 Text).

Second, countries often use fixed tariffs to cost services, while economic analysis requires complete cost breakdowns to highlight incremental effects. For this reason, unit costs and, consequently, final expected costs can vary vastly according to the specific activities chosen to define the services. The unit costs used in our analysis (Table 2) show substantial differences between countries. The UK’s unit costs are based on NHS tariffs which are heavily affected by local salaries, while Brazil’s unit costs looked at the specific activity and the time required to complete it. South Africa’s unit costs are also heavily influenced by the assumptions made about the activities involved in the service. The UK sources assume one clinic visit with a specialized nurse during the IGRA strategy and two visits in the TST one, thus considerably lowering IGRA costs compared to TST and Diaskintest in that country. Diversely, two clinic visits are assumed for both TST and IGRA when costing the strategies in South Africa [23], plus one outpatient laboratory visit to complete phlebotomy. This occurs as IGRA testing is usually only available in peripheral laboratory settings.

Third, our model represents the general population, with no co-morbidities nor TB drug-resistant strains considered. Future work focusing on scenario analysis of at-risk populations (such as HIV-positive, children and senior patients, contacts of TB cases, migrants etc.), could provide a clearer picture of the possible heterogeneity of the cost-effectiveness of TBST tests by populations. Diaskintest being the only novel skin test considered because of data availability is a limitation of this analysis; further input from manufacturers is required to include additional tests in the investigation. However, as test accuracy does not differ significantly between TBST, their costs are the only difference in the parameters used. Hence, if the unit cost of the other TBST is like Diaskintest, or below the maximum identified in our analysis, the results have potential external validity.

Fourth, we assumed that the uptake of tests for TB infection and subsequent treatment following positive results are same across different tests. However, this may not be true; for example, people may prefer tests that do not require a return visit (i.e. IGRA) or conversely those that do not require phlebotomy (i.e. skin tests). Most IGRA estimates this analysis were based on QFT, however results would unlikely differ for T-SPOT since the IGRA types perform similarly [5, 8]. Lastly, while we used the best available data on the test accuracy from a recent systematic review, there were limited data on Diaskintest. The results might differ if the accuracy is lower while it would be reasonable to assume that it performs similarly to IGRA, based on the available data and consideration of the antigen used. In addition, the accuracy of TBST may differ by setting. In the review, specificity was available only in low TB incidence countries. For instance, Diaskintest studies in Russia suggests high false-positive rate for TST likely due to repeat BCG vaccination [39]. The comparative benefit of specific tests in such settings are likely to be larger in such settings.

Our study has several strengths. We compared all the widely accepted and most used strategies and accounting for variability over the main parameters to estimate likely value for money. We compared TBST with TST and IGRA, tests that had already been shown as cost-effective strategies [34]. To our knowledge, this is the first study employing cost-effectiveness analyses using a comparative approach between country contexts while accounting for extensive sources of costs. Our study uses the most up-to-date parameters for tests’ specificity and sensitivity [5], and costs incurred and our analysis is also comparable to previous articles examining the benefits of screening for TBI [16, 34, 38]. We used QALYs to capture both the losses in quality of life and years of life gained. Our study supports and yields alternatives to presently used tests for TBI which have similar accuracy to IGRA tests and even reduced economic costs than TST.

Whilst Diaskintest was the dominant strategy in countries with a higher TB burden (Brazil and South Africa), our conclusions are based on different assumptions and still require more information to determine efficiency. Considering additional strategies, such as TBST, to the currently used tests for diagnosing TBI might help people have more manageable and more affordable access to timely preventive TB treatment. This might also serve as a starting point to expand the offer of tests in countries experiencing similar characteristics. Nonetheless, further budget impact analyses considering ease of implementation, equity and broad access to supply are still needed to adopt these strategies.

## Supporting information

S1 Text (DOCX)
