# Supplementary material for: Economic evaluation of novel Mycobacterium tuberculosis specific antigen-based skin tests for detection of TB infection: A modelling study
Source: PLOS Glob Public Health. 2023 Dec 20;3(12):e0002573. doi: 10.1371/journal.pgph.0002573 (PMC10732392; doi:10.1371/journal.pgph.0002573)
Supplement: S1 Text — (DOCX) [file pgph.0002573.s001.docx]

**Supplementary appendix**

Economic evaluation of novel *Mycobacterium tuberculosis* specific antigen-based skin tests for detection of TB infection compared to currently available tests in Brazil, UK, and South Africa: A modelling study

Table of Contents

[I. Model parameters additional information 2](#_Toc151763360)

[II. Diaskintest costs breakdown (Table D and E) 5](#_Toc151763361)

[III. Sensitivity Analysis 6](#_Toc151763362)

[IV. Drummonds Checklist for our study quality 7](#_Toc151763373)

[V. References 10](#_Toc151763374)

# **I. Model parameters additional information**

**Figure A**. Model diagram.

**
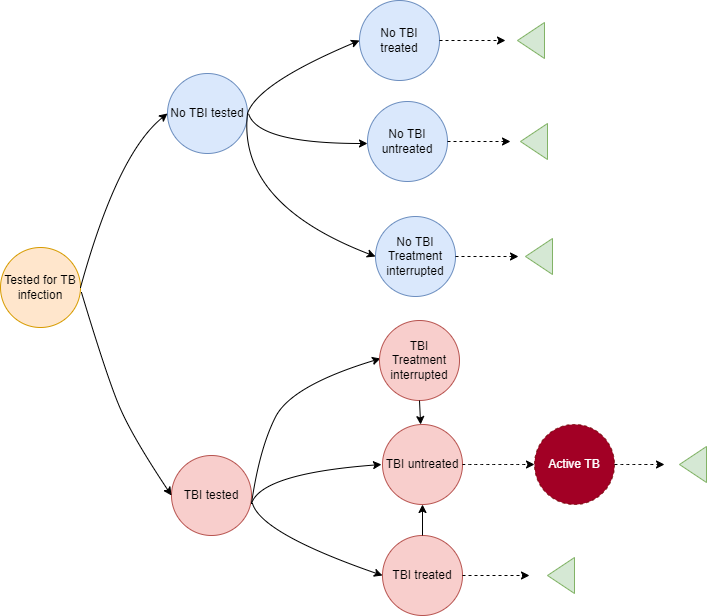
**

Notes: The model describes the following states and steps in the TB infection care cascade: (1) no tuberculosis infection, tested; (2) tuberculosis infection, tested; (3) no tuberculosis infection, treated; (4) no tuberculosis infection, untreated; (5) no tuberculosis infection, treatment started but interrupted; (6) tuberculosis infection, treated; (7) tuberculosis infection, untreated; (8) tuberculosis infection, treatment started but interrupted; (9) active TB; (10) no TB; (11) death. We start with a cohort of individuals without TB.

**Table A.** Calculation of the TB infection (TBI) progression

| **ISO code** | **LTBI estimates 2014 ^1^** | **Population estimates 2014 ^2^** | **TBI/population (%)** |
| --- | --- | --- | --- |
| BRA | 26,900,000  [19,100,000 - 36,700,000] | 202,763,744 | 13.27  [9.4 – 18.1] |
| GBR | 1,150,000  [ 867,000 - 1,790,000] | 65,423,048 | 1.76  [1.3 – 2.7] |
| ZAF | 17,200,000  [15,700,000 - 19,700,000] | 54,544,184 | 31.53  [28.8 – 36.1] |

**Table B.** Progression from TBI to active TB: population estimates

| **Progression from LTBI to active TB, by treatment received** | **Coeff probability (95% CI)** | **Source** |
| --- | --- | --- |
| **General population** |  |  |
| Evolution of recent TBI to TB, without treatment for TBI | 0.08 (0.05–0.10) | ^3-5^ |
| Evolution of remote TBI to TB, without treatment for TBI | 0.04 (0.025–0.05) | ^3-5^ |
| **HIV status** |  |  |
| Proportion of TST or IGRA positive, untreated, HIV negative individuals progressing to active TB (post‐exposure TB) over 20 years | 0.05 (0.025‐0.15) | ^6,7^ |
| Proportion of TST or IGRA positive, untreated, HIV positive individuals progressing to active TB (post‐exposure TB) over 20 years | 0.40 (0.20‐0.80) | ^6,7^ |

Notes: All individuals in the model are assumed to belong to the general population with recent TBI infection. Differentiation between HIV status or duration of infection are not included in the model but these values are reported in this table to show how the model could be adapted to study different scenarios by modifying its parametrisation.

**Table C.** Costs per TBI treatment, by country (not inflated, different currencies)

| **Cost per TBI treatment regime, by country** | **Drug costs** | **Staff costs (medical and nurse follow-up consultations) ^a^** | **Source** |
| --- | --- | --- | --- |
| Brazil | | | |
|  |  |  | 2010 costs ^8^.  Single cost per medical consultation= $4.3  Single cost Follow-up check= $4.3 |
| 3H | $36.09 | $12.9 |  |
| 6H | $72.18 | $25.8 |  |
| 9H | $108.27 | $38.7 |  |
| 12H | $144.36 | $51.6 |  |
|  |  |  |  |
| South Africa |  |  | 2016 costs,^9^.  Single cost per medical consultation= $0.98  Single cost Follow-up check= $0.77 |
|  |  |  |  |
| 3H | $2.7 | $2.52 |  |
| 6H | $5.4 | $4.83 |  |
| 9H | $8.1 | $7.14 |  |
| 12H | $10.8 | $9.45 |  |
|  |  |  |  |
| United Kingdom* |  |  |  |
|  |  |  | 2015 costs ^10^  Single cost per medical consultation= £126  Single cost Follow-up check= £64 |
| 3H | £173 | £254 |  |
| 6H | £341 | £446 |  |
| 9H | £681 | £638 |  |
| 12H | £693 | £830 |  |
|  |  |  |  |

Notes: 3H= three months of treatment with isoniazid (300 mg/day), 6H= 6 months treatment with isoniazid, 9H= = 9 months treatment with isoniazid, 12= 12 months treatment with isoniazid, 2. ^a^ Calculated considering one medical check plus the number of follow-up checks administered by nurses depending upon the regimen, it is as follows: 3H: 2 nurse consultations; 6H: 5; 9H: 8; 12H: 11.

*Drug costs were obtained from the NHS drug tariff (2014) (for H and R) and British National Formulary (2013) (for Pz); quantities of drugs used for each regimen were supplied by NICE. Staff costs were calculated from the amounts of staff time required for administration of LTBI treatment, based on GDG advice, and the cost of that time according to NHS reference costs (Curtis 2013).

# **II. Diaskintest costs breakdown (Table D and E)**

**Table D.** Cost of the Diaskintest per vial for different delivery volumes and considering that 1 vial is used for 15 patients

| Volume | Price per vial | Delivery costs  Brazil | Delivery costs  UK | Delivery costs  South Africa |
| --- | --- | --- | --- | --- |
| 667 pack | $ 24.3 | $ 7,405 | $ 5,227 | $ 5,676 |
| 6,667 pack | $ 22.3 | $ 18,560 | $ 7,948 | $ 18,199 |
| 30,000 pack | $ 19.3 | - | - | - |
| 60,000 pack | $ 19.3 | - | - | - |
| 150,000 and more pack | $ 17.8 | - | - | - |

*Notes:* All prices are indicated for 1 vial (for 15 patients). The 100% shelf life of Diaskintest is 2 years. Data provided by the test manufacturer.

**Table E.** Full costs including delivery, and cost per patient by country

| Volume | Full price excluding delivery | Number of patients treated | Full costs + delivery  Brazil | Full costs + delivery  UK | Full costs + delivery  South Africa |
| --- | --- | --- | --- | --- | --- |
| 667 pack | $16,208.1 | 10,005 | $ 7,405 | $ 5,227 | $ 5,676 |
| 6,667 pack | $148,674.1 | 100,005 | $ 18,560 | $ 7,948 | $ 18,199 |
| Price per patient: |  |  |  |  |  |
| 667 pack | $ 1.62 | - | $ 2.36 | $ 2.14 | $ 2.19 |
| 6,667 pack | $ 1.49 | - | $ 1.67 | $ 1.57 | $ 1.67 |

*Notes*. The 100% shelf life of Diaskintest is 2 years. Data provided by the test manufacturer.

# **III. Sensitivity Analysis**

# **Table F:** Maximum increase in unit cost a TBST regardless of type, compared to Diaskintest, can have to be considered cost saving and cost-effective in Brazil and South Africa, or cost-effective in UK.

| Country | Threshold increase in TBST Unit Cost |
| --- | --- |
| Brazil | $10.73 |
| South Africa | $9.33 |
| UK | $244.57 |

# **Table G:** Expected cost of Diaskintest if only one test is performed with each vial.

| Country | Diaskintest Expected Cost  (multi-dose) | Cost Saving? | Cost-Effective*? | Diaskintest Expected Cost  (Single use) | Cost Saving? | Cost-Effective*? |
| --- | --- | --- | --- | --- | --- | --- |
| Brazil | $29.84 | Yes | Yes | $38.58 | No | Yes |
| South Africa | $60.50 | Yes | Yes | $68.73 | No | Yes |
| UK | $640.71 | No | Yes | $649.59 | No | Yes |

*WTP: $7,544 (BR); $4,714 (SA); $20,223 (UK)

# **IV. Drummonds Checklist for our study quality**

We assessed the quality of our study by employing Drummond’s checklist to assess the quality of our study (Table B6).

**Table H:** Drummond checklist for the quality of our study: Cost-effectiveness analyses for Novel skin tests for diagnosing TBI

| **Drummond Checklist Questions** | **Our study** |
| --- | --- |
| **1.Was a well-defined question posed in answerable form?** |  |
| 1.1. Did the study examine both costs and effects of the service(s) or programme(s)? | Yes |
| 1.2. Did the study involve a comparison of alternatives? | Yes |
| 1.3. Was a viewpoint for the analysis stated and was the study placed in any decision-making context? | Yes |
|  | Adult population |
| **2. Was a comprehensive description of the competing alternatives given?** |  |
| 2.1. Were there any important alternatives omitted? | No |
| 2.2. Was (should) a do-nothing alternative be considered? | No |
| **3.    Was the effectiveness of the programme or services established?** |  |
| 3.1. Was this done through a randomised, controlled clinical trial? If so, did the trial protocol reflect what would happen in regular practice? | No |
| 3.2. Was effectiveness established through an overview of clinical studies? | Yes  Sensitivity and specificity of tests values from number of clinical studies and systematic reviews |
| 3.3. Were observational data or assumptions used to establish effectiveness? | Yes |
| If so, what are the potential biases in results? |  |
|  | All assumptions for effectiveness all have adequate references/reasoning. |
| **4. Were all the important and relevant costs and consequences for each alternative identified?** |  |
| 4.1. Was the range wide enough for the research question at hand? | Yes |
| 4.2. Did it cover all relevant viewpoints? | Yes |
| 4.3. Were the capital costs, as well as operating costs, included? | Diagnostic, treatment, staff, laboratory, material, and operating costs included |
|  |  |
|  |  |
| **5.    Were costs and consequences measured accurately in appropriate physical units?** |  |
| 5.1. Were any of the identified items omitted from measurement? | No |
|  |  |
| If so, does this mean that they carried no weight in the subsequent analysis? |  |
| 5.2. Were there any special circumstances (e.g., joint use of resources) that made measurement difficult? Were these circumstances handled appropriately? | No |
| **6.    Were the cost and consequences valued credibly?** |  |
| 6.1. Were the sources of all values clearly identified? | Yes |
| **6.2. Were market values employed for changes involving resources gained or depleted?** | Yes |
| 6.3. Where market values were absent (e.g. volunteer labour), or market values did not reflect actual values (such as clinic space donated at a reduced rate), were adjustments made to approximate market values? | No |
| 6.4. Was the valuation of consequences appropriate for the question posed? | No |
| **7.    Were costs and consequences adjusted for differential timing?** |  |
| 7.1. Were costs and consequences that occur in the future ‘discounted’ to their present values? | Yes |
| 7.2. Was there any justification given for the discount rate used? | Yes |
| **8.    Was an incremental analysis of costs and consequences of alternatives performed?** |  |
| 8.1. Were the additional (incremental) costs generated by one alternative over another compared to the additional effects, benefits, or utilities generated? | Yes |
| **9.    Was allowance made for uncertainty in the estimates of costs and consequences?** |  |
| 9.1. If data on costs and consequences were stochastic (randomly determined sequence of observations), were appropriate statistical analyses performed? | Yes |
| 9.2. If sensitivity analysis was employed, was justification provided for the range of values (or for key study parameters)? | Yes |
| 9.3. Were the study results sensitive to changes in the values? | Yes |
|  |  |
|  | Sensitive to treatments received. |
| **10.    Did the presentation and discussion of study results include all issues of concern to users?** |  |
| 10.1. Were the conclusions of the analysis based on some overall index or ratio of costs to consequences (e.g. cost-effectiveness ratio)? | Yes |
| 10.2. Were the results compared with those of others who have investigated the same question? If so, were allowances made for potential differences in study methodology? | Yes |
| 10.3. Did the study discuss the generalisability of the results to other settings and patient/client groups? | Yes |
| 10.4. Did the study allude to, or take account of, other important factors in the choice or decision under consideration (e.g. distribution of costs and consequences, or relevant ethical issues)? | Yes |
| 10.5. Did the study discuss issues of implementation, such as the feasibility of adopting the ‘preferred’ programme given existing financial or other constraints, and whether any freed resources could be redeployed to other worthwhile programmes? | Yes |

# **V. References**

1. Houben RM, Dodd PJ. The global burden of latent tuberculosis infection: a re-estimation using mathematical modelling. *PLoS medicine* 2016; **13**(10): e1002152.

2. World Health Organization. Global tuberculosis report 2020: World Health Organization; 2020.

3. Vynnycky E, Fine P. The natural history of tuberculosis: the implications of age-dependent risks of disease and the role of reinfection. *Epidemiology & Infection* 1997; **119**(2): 183-201.

4. Loureiro RB, Maciel ELN, Caetano R, et al. Cost-effectiveness of QuantiFERON-TB Gold In-Tube versus tuberculin skin test for diagnosis and treatment of Latent Tuberculosis Infection in primary health care workers in Brazil. *PloS one* 2019; **14**(11): e0225197.

5. Pai M, Zwerling A, Menzies D. Systematic review: T-cell–based assays for the diagnosis of latent tuberculosis infection: an update. *Annals of internal medicine* 2008; **149**(3): 177-84.

6. Horsburgh Jr CR. Priorities for the treatment of latent tuberculosis infection in the United States. *New England journal of medicine* 2004; **350**(20): 2060-7.

7. Pareek M, Bond M, Shorey J, et al. Community-based evaluation of immigrant tuberculosis screening using interferon γ release assays and tuberculin skin testing: observational study and economic analysis. *Thorax* 2013; **68**(3): 230-9.

8. Steffen RE, Caetano R, Pinto M, et al. Cost-effectiveness of Quantiferon®-TB Gold-in-Tube versus tuberculin skin testing for contact screening and treatment of latent tuberculosis infection in Brazil. *PloS one* 2013; **8**(4): e59546.

9. Kim H, Hanrahan C, Martinson N, Golub J, Dowdy D. Cost-effectiveness of universal isoniazid preventive therapy among HIV-infected pregnant women in South Africa. *The International Journal of Tuberculosis and Lung Disease* 2018; **22**(12): 1435-42.

10. White P, Jit M. What is the Cost-Effectiveness of Latent Tuberculosis Infection (LTBI) Treatment with Different Regimens. London: Imperial College Consultants; 2015.

**List of legends**

**Figure A**. Model diagram.

**Table A.** Calculation of the TB infection (TBI) progression.

**Table B.** Progression from TBI to active TB: population estimates.
**Table C.** Costs per TBI treatment, by country (not inflated, different currencies).
**Table D.** Cost of the Diaskintest per vial for different delivery volumes.
**Table E.** Full costs including delivery, and cost per patient by country.
**Table F:** Maximum increase in unit cost a TBST regardless of type, compared to Diaskintest, can have to be considered cost saving and cost-effective in Brazil and South Africa, or cost-effective in UK.
**Table G**: Expected cost of Diaskintest if only one test is performed with each vial.
**Table H**: Drummond checklist for the quality of our study: Cost-effectiveness analyses for Novel skin tests for diagnosing TBI.
